# Relationship between feed efficiency indexes and performance, body measurements, digestibility, energy partitioning, and nitrogen partitioning in pre-weaning dairy heifers

**DOI:** 10.1371/journal.pone.0223368

**Published:** 2019-10-10

**Authors:** Camila Flávia de Assis Lage, Sandra Gesteira Coelho, Hilton do Carmo Diniz Neto, Victor Marco Rocha Malacco, João Paulo Pacheco Rodrigues, João Paulo Sacramento, Fernanda Samarini Machado, Luiz Gustavo Ribeiro Pereira, Thierry Ribeiro Tomich, Mariana Magalhães Campos

**Affiliations:** 1 Department of Animal Science, School of Veterinary, Federal University of Minas Gerais, Av. Antônio Carlos, Belo Horizonte—MG, Brazil; 2 Institute of Studies of the Humid Tropic, Federal University of South and Southeast of Pará, Xinguara, Pará, Brazil; 3 Department of Bioengineering, Federal University of São João Del Rey, Praça Frei Orlando, Centro, São João Del-Rei, Minas Gerais, Brazil; 4 Brazilian Agricultural Research Corporation (Empresa Brasileira de Pesquisa Agropecuária, EMBRAPA), Embrapa Dairy Cattle, Av. Eugênio do Nascimento, Juiz de Fora—MG, Brazil; Universidade Federal de Mato Grosso do Sul, BRAZIL

## Abstract

The objectives of this study were: 1) to classify animals into groups of high and low feed efficiency using two feed efficiency indexes (Residual feed intake (**RFI**) and residual feed intake and body weight gain (**RIG**)), and 2) to evaluate if pre-weaning heifer calves divergent for feed efficiency indexes exhibit differences in performance, body measurements, digestibility, energy partitioning, and nitrogen partitioning. A total of 32 Gyr heifer calves were enrolled in a 63-d trial and classified into two feed efficiency (**FE**) groups based on RFI and RIG (mean ± 0.5 SD). The groups were classified as high efficiency (**HE**) RFI (HE RFI, n = 9; HE RIG, n = 10), and low efficiency (**LE**) RFI (LE RFI, n = 10; LE RIG, n = 11). The remaining animals were classified as intermediate (n = 13 (RFI) and n = 11 (RIG)). HE and LE calves had RFI values of—0.052 and 0.049 kg/d (P < 0.05), respectively. The HE RFI group consumed 8.9% less solid diet than the LE RFI group. HE RFI animals exhibited an increased digestibility of crude protein and ether extract and tended to have greater total dry and organic matter digestibility. LE RFI animals had greater gross energy and nitrogen intake, though greater fecal losses resulted in a tendency to reduce energy and nitrogen use efficiency. HE and LE calves had RIG values of 0.080 and -0.077kg/d (P ≤ 0.01), respectively. HE RIG animals exhibited greater average daily gain (9.4%), body weight (**BW**), and heart girth, though HE RIG group exhibited narrower hip width. HE RIG animals tended to have greater ether extract digestibility but greater methane losses (% of gross energy). HE RFI in pre-weaning heifers seems to be related to differences in digestibility. Divergent animals for RIG during the assessed phase appear to differ in body measurements, which may be related to differences in the composition of the gain.

## Introduction

The efficiency of an animal in converting feed into products is influenced by genetic, physiological, and environmental factors that result in individual variation in energy expenditure [1].The utilization of feed efficiency (FE) indexes aims to identify and select animals with great economic value. The greatest challenge in using such indexes involves determining which traits to include in the index and how to weigh them in order to maximize economic gain [2].

Residual feed intake (**RFI**), calculated by the difference between actual and expected animal feed intake is a feed efficiency (**FE**) index that is widely used in beef cattle, with the difference between animals reflecting inherent metabolic differences [3]. A previous study showed that the selection of slow-growing animals can be a problem associated with using RFI as an FE index, and have proposed the use of residual intake and body weight gain (**RIG**) as an alternative index in growing animals [4]. However, we are unaware of any existing work that has evaluated the use of RIG as an efficiency index in pre-weaning heifers and the possible impacts of this on physiological and productive parameters.

Differences in digestion and energy use can be important factors affecting FE. Lesser FE may be related to greater nutrient losses and greater methane yield. Greater maintenance costs can be also associated with high heat production by animals [5], though the importance of these factors influencing FE in the pre-weaning phase has not been determined.

Thus, the objectives of this study were: 1) to classify animals into groups of high and low FE using two FE indexes (RFI and RIG), and 2) to evaluate whether pre-weaning heifer calves divergent for FE indexes exhibit differences in performance, body measurements, digestibility, energy partitioning, and nitrogen partitioning.

## Material and methods

### Calves, housing, management, and treatments

This study was approved by the Ethics Committee of Embrapa Dairy Cattle (number: 7194210316). The experiment was conducted at the Experimental Farm of Embrapa Dairy Cattle, located in Coronel Pacheco, Minas Gerais, Brazil.

A total of 32 Gyr heifer calves produced by in vitro fertilization and born during the autumn (April to June) were used. After birth, the animals were immediately separated from their dams, weighed, and had the umbilical cord immersed in iodine solution (10%).

Colostrum was administered (10% of BW; >50g of IgG L) up to 6 hours after birth. Blood samples were collected via jugular venipuncture up to 48 hours post-birth to measure total plasma protein (g/dL) using an electronic refractometer (Serum protein REF-301, Biocotek, Beilun, Ningbo, China).

Samples were centrifuged at 1,800 x g for 10 minutes at room temperature (22–25°C). Total serum protein > 5.5 g / dL was used as a threshold for good transfer of immunity [6]. The heifers were housed in a shed without lateral walls, in individual sand beds (1.25 x 1.75 m) contained by chains 1.2 m in length.

The amount of milk offered for each heifer was based on their metabolic weight at birth. The amount of milk routinely supplied in Brazilian farms is 6 L of whole milk for animals with a mean birth weight of 35 kg [7], was used as a reference. This equates to 42% of the metabolic weight at birth in liters of milk. The objective was to standardize the amount of nutrients supplied to the heifers from the liquid diet. The mean weight of the heifers at birth was 25.2 ± 3.2 kg (mean ± SD). Consequently, the daily milk supply was 4.7 ± 0.46 L.

The volume of milk provided to the heifers was constant throughout the experiment. During the pre-weaning period, heifers received a liquid diet divided into two equal meals offered at 0700 and 1400 in nipple buckets (Milk Bar®, New Zealand). Heifers received transition milk until 3 days of age, and whole milk from the 4th to the 77th day of age. On the 78th day of age, milk supply was reduced by half and animals were weaned on the 81st day of age.

Water and solid diet were offered *ad libitum* in buckets (10% of refusals for solid diet). This solid diet was composed of 92% starter (Soymax Rumen pre-initial, 18% flocculated, Total Alimentos, Três Corações, Minas Gerais, Brazil) and 8% Tifton 85 (*Cynodon* spp) hay chopped in 5 cm length, as fed (Table 1).

**Table 1:** Nutritional composition (DM basis, % unless otherwise noted) of hay, starter, and total solid diet (TSD, 92% concentrate and 8% hay) offered to calves during the pre-weaning, from 14 to 77 days of age.

Nutritional composition	Hay	Starter	TSD^1^
DM^2^	79.4	84.3	84.0
CP^3^	10.0	19.3	18.5
OM^4^	74.0	78.8	78.4
EE^5^	3.27	3.33	3.33
NDF^6^	75.8	28.8	32.5
GE^7^ (kJ/kg)	17326	17753	17719

^1^TSD = total solid diet

^2^DM = dry matter

^3^CP = crude protein

^4^OM = organic matter

^5^EE = ether extract

^6^NDF = neutral detergent fiber

^7^GE = gross energy.

### Handling and health parameters

On the 10^th^ day of life, an oral anticocciodiostatic was administered to the animals (Isocox, Ouro Fino Saúde Animal, Cravinhos, São Paulo, Brazil) at a dose of 3 mL per kg of BW. Fecal scores were evaluated as follows: 1–normal (firm, but not hard); 2–soft (does not hold form, piles but spreads slightly); 3–runny (spreads readily to approximately 6 mm depth); and 4–watery (liquid consistency, splatters) [8]. A heifer was considered to have diarrhea if the fecal score was 3 or 4, and this condition was treated according to the farm protocol. All the episodes of diarrhea occurred within the first two weeks of life and did not influence the calculation of FE indexes.

All heifers were dehorned at 35±3 d of age using hot iron. They received local anesthesia (5.0 mL/horn, Lidovet, Bravet, Engenho Novo, Brazil) prior to the procedure and two days of non-steroid anti-inflammatory (0.025 mL/kg, Maxicam 2%, Ouro fino, Cravinhos, Brazil).

### Nutrient composition analysis

Milk samples were collected twice daily (morning and afternoon) and analyzed for total solids, crude protein (**CP**), lactose, and fat. Milk component analysis was performed using an infrared analyzer (Bentley model 2000, Bentley Instruments Inc., Chaska, MN, USA). Mean ± SD values for the milk analysis were: 12.9% ± 1.1 for total solids, 4.4% ± 1.0 for fat, 3.1% ± 0.1 for CP, and 4.5% ± 0.1 for lactose.

Samples of the solid diet (**TSD**; hay and starter) were collected three times a week and homogenized weekly in a pool. Individual refusals were collected daily and were also homogenized in weekly a pool. Samples were stored at -20° C until processing. Feed samples were oven dried at 55°C for 72 hours, ground through a 1 mm sieve in a Wiley type mill (model 3, Arthur H. Thomas Co., Philadelphia, PA, USA) and analyzed for dry matter (**DM**), CP, ether extract (**EE**), ash [9] and neutral detergent fiber (**NDF**) [10]. Gross energy was determined using an adiabatic calorimeter (IKA—C5000, IKA® Works, Staufen, Germany).

### Intake, performance, and body measurements

Milk, TSD, and water intake were measured individually. Daily intakes of milk were measured by the difference between offers and the refusals of the two meals (0700 and 1400). Water and TSD intake were calculated by the difference between offers and refusals measured 0900 daily. Feed and water were weighed using a bench scale (9094 plus, Toledo®, São Bernardo do Campo, São Paulo, Brazil) and a portable scale (WH-A04, WeiHeng, China), respectively. Scales had a precision of 0.1 g and 10 g, respectively.

Weight and body measurements were performed before the morning meal on days 3 and 7 after birth, and weekly from day 8 onward. Body weight was measured using a mobile mechanical scale (ICS-300 Móvel Mecânica, Coimma®, Dracena, São Paulo, Brazil) with precision of 0.1 kg. Withers height and hip height were measured using a measuring stick (Walmur, Porto Alegre, RS, Brazil). Hip width and heart girth were measured using a measuring tape (Bovitec, São Paulo, SP, Brazil).

### Feed efficiency indexes

Solid feed was offered since the first day of life, but feed efficiency evaluations started with 14 d of age since there was no expressive solid intake before this age. Intake and performance were evaluated from the 14^th^ to the 77^th^ day of age, and the indexes were calculated based on 63 days of observation [11].The growth rate of the animals was modeled by linear regression of BW against time over the trial duration, and the regression coefficients were calculated for the average daily gain (**ADG**) of each animal. Mean daily feed intake was calculated for each animal over the trial period and corrected for DM. The average metabolic weight (BW^0.75^) was calculated using the BW at the 46^th^ day of age, which was the middle of the test period.

Dry matter intake, BW^0.75^, and ADG were used to estimate RFI and residual body weight gain using linear regressions [3], where RFI and residual body weight gain (**RG**) were calculated as the difference between actual and predicted DMI and ADG, respectively, as follows:

$$Y_{j}=\beta_{0}+\beta_{1}(BW^{0.75}{}_{j})+\beta_{2}(ADG_{j}\;or\;DMI_{j})+e_{j},$$

where Yj is the standardized DMI (RFI) or ADG (RG) of calf j, β0 is the intercept, β1 is the regression coefficient for BW^0.75^, β2 is the regression coefficient for ADG (RFI) or DMI (RG), and ej is the error term for calf j.

In the present study, RG was not used as an FE index. The RG calculation was performed to estimate RIG.

To calculate RIG, the residues for RFI and RG were added as [4]:

RIG=[RFIx(‐1)]+RG

Based on these indexes, the animals were classified into four groups: high efficiency (**HE**) and low efficiency (**LE**) for RFI and RIG. HE indicated RFI < 0.5 SD below the mean (n = 9) and RIG > 0.5 SD above the mean (n = 10), while LE indicated RFI > 0.5 SD above the mean (n = 10) and RIG < 0.5 SD below the mean (n = 11). The remaining animals were classified as intermediate and were not included in subsequent analyses. The feed efficiency indexes, DMI, BW, and ADG of the high and low efficiency groups are presented in Table 2.

**Table 2:** Intake, performance and body measurements in pre-weaning calves (14 to 77 days old) classified as high efficiency (HE) and low efficiency (LE) for RFI and RIG.

	RFI^1^			*P*-value	RIG^2^			*P*-value
Item	HE^3^	LE^4^	SEM	G^5^	HE	LE	SEM	G
RFI	-0.052	0.049	0.01	<0.01				
RIG					0.080	-0.077	0.018	<0.01
*Intake (kg/d)*								
Water	0.33	0.38	0.07	0.67	0.35	0.38	0.03	0.41
Solid diet	0.16	0.24	0.02	0.001	0.17	0.20	0.01	0.07
Milk	0.61	0.60	0.003	0.07	0.60	0.61	0.001	0.61
Total	0.76	0.84	0.02	0.002	0.78	0.80	0.01	0.08
*Body measurements*								
ADG^6^ (kg/d)	0.60	0.59	0.02	0.67	0.60	0.55	0.02	0.04
Whither height (cm)	84.5	85.2	0.40	0.21	84.5	84.7	0.23	0.46
Hip height (cm)	88.5	88.9	0.50	0.57	88.2	88.5	0.22	0.37
Body weight (kg)	48.2	46.6	0.77	0.16	47.8	46.3	0.25	<0.001
Hip width (cm)	22.2	22.6	0.13	0.06	22.0	22.5	0.10	<0.001
Heart girth (cm)	79.3	78.3	0.48	0.17	79.1	77.8	0.17	<0.001

^1^RFI = Residual feed intake

^2^RIG = Residual intake and gain

^3^HE = High efficiency

^4^LE = Low efficiency

**Fig 1:**
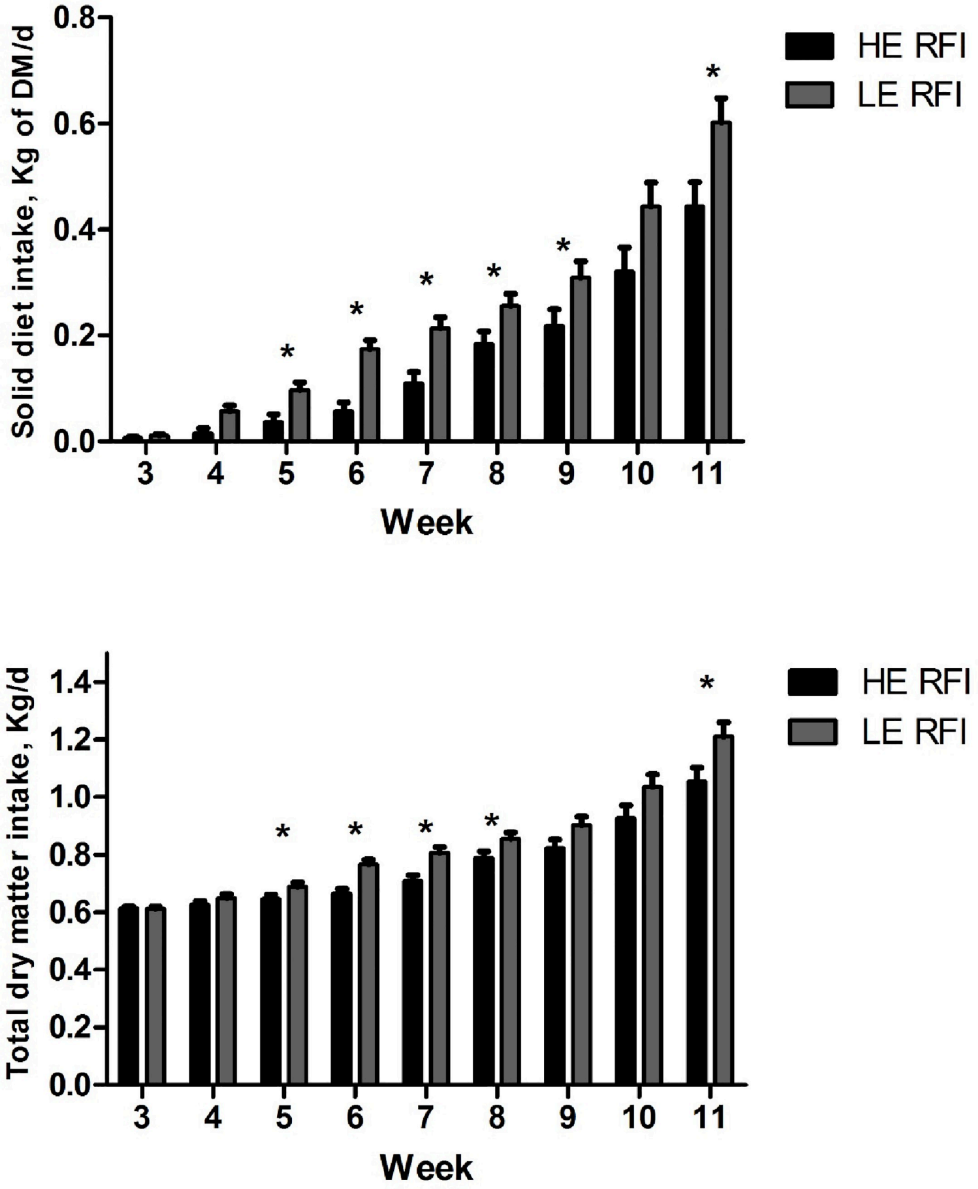
Weekly solid diet intake and total dry matter intake in pre-weaning calves (14 to 77 days old) classified as high efficiency (HE) and low efficiency (LE) for RFI.

**Fig 2:**
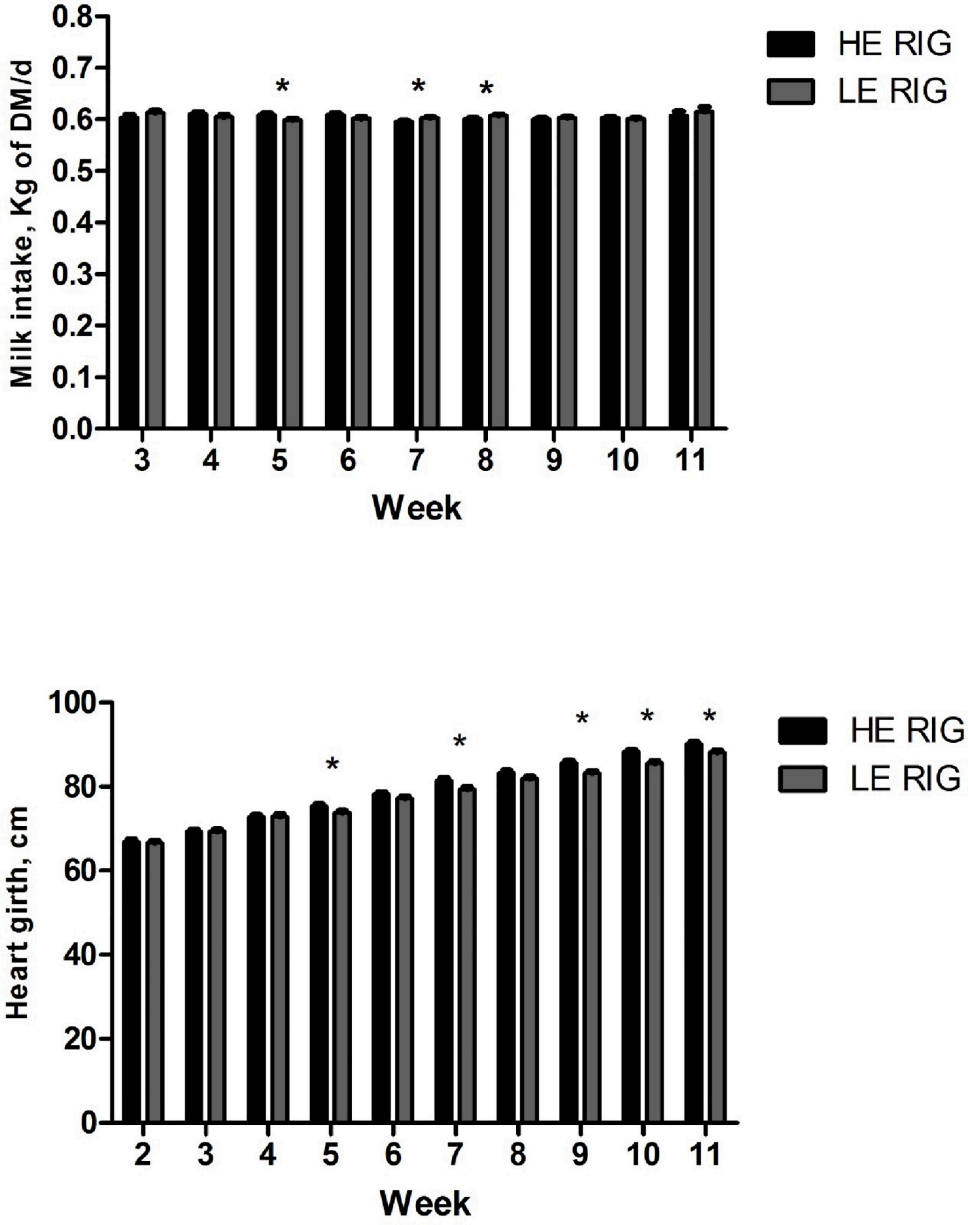
Weekly milk intake and heart girth in pre-weaning calves (14 to 77 days old) classified as high efficiency (HE) and low efficiency (LE) for RIG.

^5^G = Main effect of group. Group × period interaction, *P* ≥ 0.06, except solid diet intake and total intake for RFI groups (*P* < 0.01) and milk intake and heart girth for RIG groups (*P* < 0.05) (Figs 1 and 2, respectively)

^6^ADG = Average daily gain

### Whole tract digestibility

From the 50^th^ to the 55^th^ day of age, collections of fecal and urine were performed. They were housed in metabolic cages (dimensions of 1.50 m × 0.80 m; Intergado Ltda., Contagem, Brazil) for two consecutive days for urine and feces collection. The urine tray was designed with inclination to drain urine into 5 L containers stored in expanded polystyrene thermal boxes with ice. The volumes, weights, and urine densities of each animal were measured every 24 hours, and one 50 mL pure urine sample was taken after being filtered through cheesecloth and stored at -20°C.

After 2 days in metabolic cages, the animals were transferred into tie-stalls with a rubber mat for another 3 days of fecal collection. During these 5 days, the total fecal excretions of the animal were collected and weighed at least three times per day. Equivalent quantities of the daily sub-samples were combined into one sample per animal.

Samples of starter, hay, and orts were also collected daily, during the 5 days, from each animal. At the end of the collection period, equivalent quantities of the daily sub-samples were combined into one sample per animal and frozen at -20°C for further analysis. After thawing, feed, orts, and feces samples were oven dried at 55°C for 72 hours, ground through a 1 mm sieve in a Wiley type mill (model 3, Arthur H. Thomas Co., Philadelphia, PA), and analyzed for DM, CP, EE, ash, NDF, and gross energy (as explained in the nutrient composition analysis section). Urine nitrogen content was obtained by the Kjeldahl method [9], and gross energy was determined using an adiabatic calorimeter (IKA—C5000, IKA® Works, Staufen, Germany).

### Respiratory exchanges and methane emission

On the 55^th^ (±6) day of age, respiratory exchanges and methane emissions were measured using an open-circuit respirometry chamber (Intergado® Ltda., Contagem, MG, Brazil) with a volume of 6.39 m^3^ (2.48× 1.48 × 1.74 m) made from aluminum and transparent polyethylene terephthalate glycol (PETG) walls.

Animals were housed in a metabolic cage (dimensions of 1.50 mx 0.80 m; Intergado Ltda., Contagem, Brazil) allocated inside the respirometry chamber. The air inside the chamber was maintained at 60% RH and 20°C. A mass flow meter (Flow Kit model FK-500, Sable International Systems, Las Vegas, NV) continuously pulled air from the chamber (100 L/min). Air from the chamber and ambient air were analyzed for a gas analysis and data acquisition system (13) to monitor O_2_, CO_2_, and CH_4_ concentrations by the analyzers FC-10 oxygen, CA-10 carbon dioxide and MA-10 CH_4_ (Sable International Systems, Las Vegas, NV). Total gas exchange for O_2_, CO_2_, and CH_4_ were calculated [12].

The same procedure applied for the digestibility trial was used to calculate the DMI inside the chamber. The animals were weighed before entering the chamber, and the urine volume produced inside the chamber was recorded.

### Calculations

The dry matter intake of each nutrient was calculated as the sum of intake (as fed) of each of the supplied components (milk, starter, and hay) and their respective content of DM and nutrients, discounting the quantities of DM and nutrients obtained from the orts of the milk and TSD.

Apparent digestibility values (%) were determined using the amount consumed and the amount of each nutritional component recovered in feces. Nitrogen balance was calculated by the difference between nitrogen intake in the diet and nitrogen excreted in feces and urine.

Gross energy intake (**GEI**) was calculated by the difference between the gross energy (**GE**) content of each of the supplied components (milk, starter, and hay) and those obtained in orts. GE content of milk was calculated as [13]: GE (Mcal/kg milk) = (0.0911 x % fat) + (0.0586 x % protein) + (0.0395 x % lactose).

Digestible energy intake (**DEI**) was calculated by the difference between GEI and fecal energy excretion. Subsequently, metabolizable energy intake (**MEI**) was calculated as the difference between DEI and the sum of urine energy and CH_4_ energy, which was assumed to be 39.5 kJl/L [14]. Heat production (**HP**; kJ/d) was determined based on measurements of O_2_ consumption (L/d), CO_2_, and CH_4_ production (L/d), and urine N output (g/d) applying the equation of Brouwer [14]. Energy balance (**EB**) was calculated as the difference between MEI and heat production. Percentages of energy loss through feces, urine, methane, and heat (% GEI), as well as the relationships between ME/GE, DE/GE, HP/ME, and EB/ME were calculated and used as indicators of energy efficiency.

### Statistical analyses

The statistical analyses were performed using SAS software [15]. To evaluate the effects of efficiency in the groups, the MIXED procedure was used for intake, performance, and body measurements, according to the model:

$$Y_{ijk}=\beta_{0}+\beta_{1}A_{ij}+\beta_{2}B_{ij}+G_{i}+M_{k}+{GM}_{ik}+\delta_{ij}+\varepsilon_{ijk}$$

where *Y*_*ijk*_ is the dependent variable; *β*_0_ is the intercept; *β*_1_*A*_*ij*_ is the regression coefficient for the covariate initial BW; *β*_2_*B*_*ij*_ is the regression coefficient for the covariate total serum protein; *G*_*i*_ is the fixed effect of efficiency group (RFI or RIG); *M*_*k*_ is the fixed effect of repeated measure (day or week); *GM*_*ik*_ is the fixed effect of interaction between group and repeated measure; *δ*_*ij*_ is the random error between animals within treatment; and *ε*_*ijk*_ is the random error between measurements among animals. The best covariance structure for repeated measures was chosen by the lowest corrected Akaike information criteria. The covariance structures evaluated were: variance components, composed symmetry, heterogeneous composed symmetry, autoregressive, heterogeneous autoregressive, and unstructured. For most of the dependent variables the heterogeneous composed symmetry structure was selected. For significant interaction between group and repeated measure, the differences among groups within measures were evaluated using the SLICE statement.

To evaluate data on digestibility, gas exchange, HP, energy intake, energy losses, energy use efficiency, and nitrogen balance, the following model was used:

$$Y_{ijk}=\beta_{0}+\beta_{1}A_{ij}+\beta_{2}B_{ij}+G_{i}+\varepsilon_{ijk}$$

where *Y*_*ijk*_ is the dependent variable; *β*_0_ is the intercept; *β*_1_*A*_*ij*_ is the regression coefficient for the covariate initial BW; *β*_2_*B*_*ij*_ is the regression coefficient for the covariate total serum protein; *G*_*i*_ is the fixed effect of efficiency group (RFI or RIG); and *ε*_*ijk*_ is the random error. Pearson correlation coefficients between the response variables and RFI and RIG were obtained with PROC CORR. Significance of the effects was declared at *P* ≤ 0.05 and tendency was accepted when 0.05 < *P* ≤ 0.10. Covariates were removed from the model when not significant.

## Results

### Intake and performance–Residual feed intake

Divergence was observed among the animals for RFI during the pre-weaning phase (Table 2). The average RFI for the HE group was -0.052 kg/d and was 0.049 kg/d for the LE group. HE RFI animals consumed 8.9% less than LE RFI animals.

Treatment × week interaction was observed for solid diet intake and total intake (Table 2; Fig 1). Solid diet intake was greater (*P* ≤ 0.05) for the LE RFI group from weeks 4 to 11, except for week 10 (*P* = 0.06). Total diet intake was greater (*P* ≤ 0.05) for the LE RFI group from weeks 5 to 11, except for weeks 9 and 10 (*P* = 0.07 and *P* = 0.09, respectively).

No differences in water intake were observed, though there was a tendency for greater milk intake among HE RFI animals (*P* = 0.07).

No differences in BW, ADG, withers height, hip height, and heart girth were observed between the groups (*P* ≥ 0.16). However, there was a tendency for greater (*P* = 0.06) hip width among LE RIF animals.

### Digestibility–Residual feed intake

HE RFI animals exhibited an increased digestibility of CP (*P* = 0.02) and EE (*P* = 0.02) and tended to have improved total DM (*P* = 0.06) and organic matter (**OM**) (*P* = 0.07) digestibility. No differences in NDF digestibility were observed (Table 3).

**Table 3:** Digestibility (%) of DM, OM and nutrients in pre-weaning calves (50 to 55 days old) classified as high efficiency (HE) and low efficiency (LE) for RFI and RIG.

Item	RFI^1^			*P*-value	RIG^2^			*P*-value
	HE^3^	LE^4^	SEM	G^5^	HE	LE	SEM	G
DM^6^	89.2	85.8	1.24	0.06	88.1	87.4	1.28	0.70
OM^7^	91.1	88.0	1.15	0.07	90.2	89.5	1.20	0.68
CP^8^	91.8	87.8	1.16	0.02	90.8	88.5	1.18	0.18
NDF^9^	52.7	40.5	7.56	0.27	48.0	45.7	6.37	0.80
EE^10^	96.4	93.1	0.95	0.02	96.3	93.9	0.90	0.07

^1^RFI = Residual feed intake

^2^RIG = Residual intake and gain

^3^HE = High efficiency

^4^LE = Low efficiency

^5^G = Main effect of group

^6^DM = dry matter

^7^OM = organic matter

^8^CP = crude protein

^9^NDF = neutral detergent fiber

^10^EE = ether extract

### Energy intake, losses and energy use efficiency—Residual feed intake

Positive correlations were observed between RFI and GEI (r = 0.50; *P* = 0.03), indicating that high RFI values (LE RFI) are correlated with greater gross energy intake. HE RFI animals also exhibited lesser GEI but similar DE, ME, and NE intake (Table 4).

**Table 4:** Energy intake, losses and energy use efficiency in pre-weaning calves (50 to 55 days old) classified as high efficiency (HE) and low efficiency (LE) for RFI and RIG.

Item	RFI^1^			*P*-value	RIG^2^			*P*-value
	HE^3^	LE^4^	SEM	G^5^	HE	LE	SEM	G
*Energy intake (kJ/d/BW*^*0*.*75*^*)*								
Gross energy	1000	1112	3.30	0.03	992	1063	3.56	0.17
Digestible energy	925	996	3.10	0.11	912	975	2.85	0.12
Metabolizable energy	908	979	3.10	0.12	891	958	2.80	0.11
Energy balance	305	356	3.22	0.28	290	336	2.85	0.25
*Energetic outputs (% GE)*								
Feces	7.66	10.6	1.06	0.06	8.22	8.35	0.85	0.91
Methane	0.17	0.22	0.08	0.66	0.22	0.11	0.04	0.05
Urine	1.36	1.39	0.13	0.86	1.39	1.37	0.14	0.92
Heat	60.9	56.1	2.26	<0.001	61.3	59.1	2.42	0.51
Energy balance	30.0	31.6	2.33	0.61	28.8	31.1	1.89	0.40
*Energy use efficiency (%)*								
DE:GE^6^	92.3	89.4	1.06	0.06	91.9	91.9	0.85	0.91
ME:GE^7^	90.8	87.8	1.10	0.06	90.2	90.2	0.84	1.00
HP:ME^8^	67.0	64.0	2.50	0.39	68.0	65.4	2.26	0.42
EB:ME^9^	33.0	36.0	2.50	0.39	32.0	34.6	2.26	0.42

^1^RFI = Residual feed intake

^2^RIG = Residual intake and gain

^3^HE = High efficiency

^4^LE = Low efficiency

^5^G = Main effect of group

^6^DE:GE: Ratio between digestible energy and gross energy

^7^ME:GE: Metabolizability

^8^HP:ME Ratio between heat production and gross energy

^9^EB:ME = Ratio between energy balance and gross energy

Energy losses in feces were also positive correlated with RFI (r = 0.41; *P* = 0.08). LE RFI animals had a greater DMI but tended to have greater fecal losses (*P* = 0.06). No differences in energetic losses in urine and methane emissions were observed between HE and LE RFI animals. HE RFI animals exhibited greater (*P* < 0.001) HP as a percentage of GEI.

HE RFI animals tended to have greater DE:GE (%) and ME:GE (*P* = 0.06) though with the same EB (% GE) when compared to LE RFI animals.

### Nitrogen balance–Residual feed intake

Greater (*P* = 0.05) nitrogen intake and greater (*P* = 0.02) nitrogen loss in feces were observed in LE RFI animals. However, no differences were observed in urine nitrogen losses and retained nitrogen between the groups (P ≥0.50) (Table 5).

**Table 5:** Nitrogen balance (g/d/BW^0.75^) in pre-weaning calves (50 to 55 days old) classified as high efficiency (HE) and low efficiency (LE) for RFI and RIG.

Item	RFI^1^			*P*-value	RIG^2^			*P*-value
	HE^3^	LE^4^	SEM	G^5^	HE	LE	SEM	G
*Nitrogen Balance (g/d/BW*^*0*.*75*^*)*								
Nitrogen intake	1.56	1.74	0.05	0.03	1.55	1.66	0.06	0.21
Feces nitrogen	0.13	0.21	0.02	0.01	0.15	0.19	0.02	0.14
Urine nitrogen	0.46	0.54	0.06	0.33	0.47	0.47	0.05	0.97
Retained nitrogen	0.97	0.99	0.07	0.90	0.93	0.99	0.07	0.50

^1^RFI = Residual feed intake

^2^RIG = Residual intake and gain

^3^HE = High efficiency

^4^LE = Low efficiency

^5^G = Main effect of group

### Gas exchange and HP–Residual feed intake

No differences in gas exchange (VO_2_, VCO_2_, and VCH_4_) were observed between the HE and LE RFI groups (Table 6). In addition, no differences in HP (kJ/BW^0.75^) were observed between the groups.

**Table 6:** Gas exchange and heat production in pre-weaning calves (55 ± 6 days old) classified as high efficiency (HE) and low efficiency (LE) for RFI and RIG.

Item	RFI^1^			*P*-value	RIG^2^			*P*-value
	HE^3^	LE^4^	SEM	G^5^	HE	LE	SEM	G
VO_2_^6^ (L/day/kg^0.75^)	29.0	30.3	0.82	0.27	29.0	30.2	0.67	0.24
VO_2_ (L/day)	568	567	15.2	0.95	559	554	11.0	0.75
VCO_2_^7^ (L/day/kg^0.75^)	27.3	28.0	0.57	0.39	27.4	27.9	0.49	0.50
VCO_2_ (L/day)	534	525	13.2	0.59	529	513	10.8	0.32
VCH_4_^8^ (L/day/kg^0.75^)	0.04	0.06	0.02	0.37	0.05	0.03	0.02	0.45
VCH_4_ (L/day)	0.67	1.21	0.42	0.35	0.89	0.58	0.31	0.47
VCH_4_ (L/ kg of DMI of TSD^9^)	0.93	1.77	0.56	0.30	1.27	0.93	0.45	0.60
HP^10^ (kJ/ kg^0.75^)	586	628	16.7	0.23	586	628	12.6	0.25
HP (kJ/d)	1184	1175	293	0.86	1163	1151	209	0.61

^1^RFI = Residual feed intake

^2^RIG = Residual intake and gain

^3^HE = High efficiency

^4^LE = Low efficiency

^5^G = Main effect of group

^6^VO_2_ = Oxygen volume

^7^VCO_2_ = Carbon dioxide volume

^8^VCH_4_ = Methane volume

^9^TSD = Total solid diet

^10^HP = Heat production

### Intake and performance–Residual intake and body weight gain

There was a tendency for greater solid intake (*P* = 0.07) and total intake (*P* = 0.08) in the HE RIG group compared to the LE RIG group. Moreover, an interaction was identified between treatment and week for milk intake (Table 2, Fig 2). The HE RIG group exhibited greater milk intake in the 5^th^ week (*P* = 0.02). HE RIG animals had greater ADG and BW during the study period. Notably, there was a treatment x week interaction for heart girth (Table 2, Fig 2). Differences in heart girth were observed between week 5 to week 11, except in weeks 6 and 8 (*P* = 0.015; *P* = 0.07, respectively). HE RIG animals had lesser hip width during the study period, though no differences were observed for withers height and hip height.

### Digestibility—Residual intake and body weight gain

No differences in DM, OM, CP, and NDF digestibility were identified. HE RIG animals tended to have greater EE digestibility (*P* = 0.07) when compared to LE RIG animals (Table 3).

### Energy intake, losses, and energy use efficiency—Residual intake and body weight gain

No differences in energy intake (kJ/d/BW^0.75^) and energy losses (% GE) by feces, urine, and heat were identified between HE and LE RIG animals. HE RIG animals had greater losses (% GE) by methane emissions, though no differences in energy use efficiency were found (Table 4).

### Nitrogen balance–Residual intake and body weight gain

No differences in nitrogen balance (g/d/BW^0.75^*)* between LE and HE RIG animals were observed (Table 5).

### Gas exchange and heat production–Residual intake and body weight gain

No differences were observed in gas exchange (VO_2_, VCO_2_, and VCH_4_) between HE and LE RIG groups (Table 6). In addition, no differences in HP (kJ/BW^0.75^) were identified.

## Discussion

### Residual feed intake

FE divergence tests are applicable to pre-weaning calves, as divergence in RFI and RG were observed during the rearing phase (16). Animals classified as HE and LE for RFI had differences in TSD intake, which were related to differences in starter intake. Since milk is normally fixed in dairy systems, we decide to use this system during the evaluation. Milk supply was standardized in relation to metabolic weight at birth to reduce the chance of giving different amount of nutrients between the animals. Despite this, there was a tendency towards greater milk intake in HE RFI animals, which can be attributed to differences in BW between HE and LE RFI animals over the experiment. Because milk supply was based on metabolic weight, differences in body weight may have influenced milk intake tendencies.

The total difference in intake was 8.9% between the HE and LE groups for RFI, which was lesser than that observed by previous study [16] which reported a difference of 13% for crossbreed heifers (Holstein x Gyr) during the pre-weaning phase. Starter intake during the entire trial was also lesser than observed in previous trials conducted by our research team [7,16,17]. Differences in frame size (BW at birth: 25.2 ± 3.2 kg [mean ± SD]) may have influenced these results. Although the absolute values of DMI were lesser than those reported in previous studies, when data is viewed as % of BW, Gyr calves intake is similar to crossbred and pure Holstein calves [7,16,17].

Treatment x week interactions for TSD and starter intake indicated differences in intake between HE and LE RFI groups from the third week of age, when solid diet intake in pre-weaning heifers becomes expressive [18]. Results observed in the present trial suggests that variation in DMI are observed because pre-weaning phase. Recent study, reviewing biological factors related to RFI, showed that differences in intake behavior often explains part of the differences, but factors affecting differences in feeding behavior are not yet fully understood [19]. In addition, previous experiment evaluated the within-animal repeatability of intake, growth, and FE in different feed conditions (pasture and confinement) and concluded that DM intake, and to a lesser extent RFI, were somewhat repeatable traits [20]. Feeding behavior is determined by the integration of central and peripheral signals in brain feeding centers [21]. Notably, a study that evaluated hypothalamic metabolomic profiling in cattle with divergent RFI suggested that there are differences between HE and LE groups that may be related to differences in the central regulation of intake[22]. Differences in meal patterning appears to be related to differences in RFI during pre-weaning phase.

There were no differences in water intake between the HE and LE groups for RFI despite its positive relationship with DMI [16,23]. The water intake observed in the present study was lesser than that observed by previous studies [16,17]. This could potentially be related to the season, because the present study was conducted during the colder autumn and winter, when water intake becomes lesser. Such differences among studies can also be related to the animal’s breed, because we are unaware of any study that evaluated water intake in pre-weaning Gyr heifer calves.

There were no differences in body measures, except a tendency for greater hip width in LE RFI heifers. Little is known about the relationship between RFI and body measurements. Most studies on growing animals have been performed in beef cattle, and no differences were observed in body structure between the HE and LE groups for RFI [24–27,28]. In addition, a study observed that the phenotypic correlation between RFI and ADG or body size was close to zero, indicating that selection based on RFI would not affect growth or body size [27].

HE RFI animals exhibited increased CP and EE digestibility and tended to have improved DM and OM digestibility. Pre-weaning dairy calves ingest a high protein and fat diet with a low proportion of fiber, which may explain why we did not observe differences in NDF digestibility.

It is known that as level of feed intake relative to maintenance increases, the digestion of feed tends to decrease. However, over and above systematic variation due to the amount of feed eaten, there is also genetic variation in the total tract digestion of feed [5].

Previous studies on beef steers [29], and lactating dairy cows [30] have demonstrated that animals classified as HE RFI have improved digestibility. Experiment also determined that nutrient digestibility was moderately repeatable across different diets [30]. Research conducted on pigs selected for RFI noted greater digestibility in HE RFI animals, and hypothesized that such findings could be related to the increased activity of intestinal microbial populations associated with the greater gene expression levels of intestinal nutrient transporters [31,32]. We suggest that greater digestibility found in pre-weaning heifers can also be related to greater intestinal activity, because the intestinal contribution to total tract digestion during this growth phase is high; however, more studies should be made to confirm this hypothesis.

Positive correlations were observed between RFI and GEI (r = 0.502; *P* = 0.028), indicating that LE RFI had a greater gross energy intake. Despite this, energy losses in feces were also positive correlated with RFI (r = 0.41; *P* = 0.08). Overall, while LE RFI animals eat more, they have greater fecal losses due to lesser digestibility, which results in similar DEI.

Positive correlations were also found between RFI and nitrogen intake (r = 0.50; *P* = 0.03) and losses in feces (r = 0.6; *P* = 0.005). This can be attributed to the differences in CP digestibility, because LE RFI animals had a greater intake but lowest CP digestibility, resulting in similar retained nitrogen values when compared with HE RFI group. In terms of energy intake, GEI was greater in the LE RFI group, but digestible and metabolizable intake were the same between HE and LE animals. This can be attribute to greater digestibility in the HE RFI group. When we evaluate the losses (% GE), a tendency was found to greater fecal losses in LE RFI animals; however, no differences in methane losses (% GE) and methane production parameters between HE and LE RFI were found among pre-weaning heifers in this trial. No differences in HP (kJ/kg^0.75^ and kJ/d) between the groups were observed, although heat losses (% GE) were greater for HE RFI animals. Overall, the efficiency of using metabolizable energy was the same between HE and LE RIF animals, which is evident from the HP:ME and EB:ME energy ratio (Table 4).

A previous study, that used a face mask method to evaluate methane production in pre-weaning heifers [16] did not observe significant methane production during this phase. Pre-weaned heifers do not have a fully developed rumen and also eat a grain-heavy diet that makes methane production a minor source of energy loss in these animals. Additionally, even in animals with a developed rumen, lesser methane production is not always observed in HE RFI animals. The reduction of feeding level generally increases the mean retention time of digesta in the rumen [33], which can increase methane production. Moreover, HE RFI animals often have greater total tract DM digestibility, which implies a greater amount of substrate available for fermentation and methanogenesis per unit feed [34].

### Residual intake and body weight gain

The use of RFI as an FE index can rank slow-growing animals such as HE RFI [4]. This is not applicable for the dairy industry due to the fact that slow-growing animals may take longer to achieve puberty and thus have a greater age at first calving.

A previous study proposed the use of RIG as an alternative index [4] because it can identify animals that grow rapidly and eat proportionally less than expected. The observed differences in intake and performance between animals classified as HE RIG and LE RIG in this trial met the purpose for the index, because HE RIG animals exhibited greater ADG and BW during the efficiency test. HE RIG animals also had greater heart girth, which was somewhat expected because heart girth has a high correlation with BW [35] HE RIG animals also seem to have narrow hip width compared to LE RIG animals.

Differences in body measurements such as hip width can indicate differences in phenotypic and functional features in animals [36]. A previous study noted a positive correlation between hip width and energy-corrected milk and a negative correlation between hip width and productive life, which suggests that narrow animals are more prone to be less productive, and thus more likely to be culled. Thus, the impacts of RIG utilization for selecting productive animals must be further investigated.

Interaction between treatment x week was observed for milk intake between RIG groups. Differences in milk intake between the groups are result of slightly fluctuations in milk intake in individual animals. In week 5 the animals were subjected to a dehorn process. Although we adopted practices to reduce the stress associated to dehorn procedure, individuals can have different responses to stress, which affected the milk intake in some animals. Because the present experiment is an efficiency assay, the real intake of the animals over the 63 days were computed.

No differences in DM, OM, CP, and NDF digestibility were observed, although HE RIG animals tended to have greater EE digestibility when compared to LE RIG animals. Greater efficiency in fat absorption can partially explain the greater ADG of these animals, because fat is an energy-dense nutrient.

No differences were found in energy intake, energy losses, and energy use efficiency, nitrogen balance, gas exchange, and HP between HE and LE RIG animals, with the exception of high methane loss (% GE) in HE RIG animals.

As previously discussed, the methane emissions in pre-weaning phase is low. In addition, the variation in methane production between animals was high during the study period. Due to the high variability and consequently high SE, there were no differences in daily methane production—even when considering the metabolic weight and intake of the animals.

The use of methane units as a percentage of losses normalized the data distribution and made it possible to observe the difference in methane production between the groups, with losses being proportionally greater for the HE RIG group. In the present study, the greater methane losses in the HE RIG group may have been related to the quantity of substrate available for fermentation and methanogenesis (35).

### Final considerations

The use of RFI and RIG indexes resulted in groups with different characteristics and it should be taken into account in future research and animals selection programs. Digestibility of DM, OM, CP, and EE were factors that most heavily impacted differences in RFI between pre-weaning calves. Despite the lesser intake of GE and nitrogen, RFI animals had the same energy and nitrogen retention among groups, which resulted in the same ADG. Then, intake had greater weight when grouping the calves by RFI. However, when grouping by RIG the divergency was more associated to body measurements, which may be related to differences in the composition of gain. Future research should evaluate how the use of different indexes would impact in breeding programs and which of these calves’ characteristics are associated to its efficiency indexes when cows.
